# Loss of *PINK1* enhances neurodegeneration in a mouse model of Parkinson's disease triggered by mitochondrial stress^☆^

**DOI:** 10.1016/j.neuropharm.2013.10.009

**Published:** 2014-02

**Authors:** Nicoleta Moisoi, Valentina Fedele, Jennifer Edwards, L. Miguel Martins

**Affiliations:** a Cell Death Regulation Laboratory, MRC Toxicology Unit, Hodgkin Building, Lancaster Road, Leicester LE1 9HN, UK; b Cell Physiology and Pharmacology Department, University of Leicester, Maurice Shock Building, University Road, Leicester LE1 9HN, UK

**Keywords:** Mitochondria quality control, Neurodegeneration, Parkinson's disease

## Abstract

Parkinson's disease (PD) shows a complex etiology, where both genetic and environmental factors contribute to initiation and advance of pathology. Mitochondrial dysfunction and mutation of genes implicated in mitochondria quality control are recognized contributors to etiopathology and progression of PD. Here we report the development and characterization of a genetic mouse model of PD with a combined etiology comprising: 1) induction of mitochondrial stress achieved through the expression of a mitochondrial matrix protein that accumulates in an unfolded state and 2) deletion of *PINK1* gene. Using this model we address the role of *PINK1* in mitochondrial quality control and disease progression.

To induce mitochondrial stress specifically in catecholaminergic neurons we generated transgenic animals where the conditional expression of mitochondrial *unfolded ornithine transcarbamylase* (*dOTC*) is achieved under the tyrosine hydroxylase (*Th*) promoter. The mice were characterized in terms of survival, growth and motor behaviour. The characterization was followed by analysis of cell death induced in dopaminergic neurons and responsiveness to l-dopa. We demonstrate that accumulation of dOTC in dopaminergic neurons causes neurodegeneration and motor behaviour impairment that illustrates a parkinsonian phenotype. This associates with l-dopa responsiveness validating the model as a model of PD. The combined transgenic model where dOTC is overexpressed in *PINK1* KO background presents increased neurodegeneration as compared to dOTC transgenic in wild-type background. Moreover, this combined model does not show responsiveness to l-dopa. Our *in vivo* data show that loss of *PINK1* accelerates neurodegenerative phenotypes induced by mitochondrial stress triggered by the expression of an unfolded protein in this organelle.

## Highlights

• We developed a novel genetic mouse model of Parkinson's disease (PD).
• The model has a combined aetiology: 1) mitochondrial stress and 2) deletion of *PINK1*.
• Unfolded proteins in mitochondria of dopaminergic neurons cause PD phenotypes.
• Loss of *PINK1* accelerates PD phenotypes induced by mitochondrial stress.

## 1 Introduction

Parkinson's disease (PD) is the second most common neurodegenerative disorder, with a prevalence second only to that of Alzheimer's disease. The primary hallmarks of the disease are degeneration of multiple neuronal types including, most notably, dopaminergic (DA) neurons in the Substantia Nigra of the midbrain (Shulman et al., 2011), and formation of Lewy bodies, intracytoplasmic inclusions that are mainly composed of fibrillar α-synuclein. The, pathology of many non-dopaminergic neurons including olfactory and brain stem neurons precedes that of DA neurons (Braak et al., 2003). Patients with PD present characteristic motor symptoms, such as resting tremor, slowness of movement, rigidity, postural instability, and gait perturbation. PD patients also present a combination of non-motor symptoms including psychiatric symptoms such as depression and anxiety, autonomic dysfunction (involving cardiac and digestive systems), perturbed sleep patterns, and musculoskeletal abnormalities (Simuni and Sethi, 2008). PD has a complex multifactorial etiology where both environmental and genetic factors appear to be important.

Mitochondrial dysfunction is at the core of several age related neurodegenerative diseases including Parkinson's disease (Lezi and Swerdlow, 2012). Over the last years mutations found in familial or sporadic cases of neurodegenerative disorders provided insights into the mechanisms underlying neurodegeneration. That is also the case for PD where a number of proteins have been found mutated and their function has been directly or indirectly correlated with mitochondria, oxidative stress and accumulation of unfolded proteins (Kumar et al., 2012). The presence of protein aggregates in neurodegeneration, including PD, has been well documented. In PD, Lewy bodies are known to contain unfolded proteins of mitochondrial origin, together with proteins that are normally located in the cytosol or at neuronal synapses. Although the accumulation of unfolded proteins in the mitochondria has been debated (de Castro et al., 2010) there is now clear evidence both from patient samples and animal models that in PD there is accumulation of mitochondrial unfolded proteins correlated with mitochondrial stress signalling (de Castro et al., 2012).

Cells have developed several defence mechanisms to cope with mitochondrial damage (Tatsuta and Langer, 2008; Baker et al., 2011). Molecular quality control represents the first step in the mitochondrial defence mechanisms. This implicates the up-regulation of nuclear genes encoding for mitochondrial chaperones and proteases to assist the removal of misfolded and non-assembled polypeptides. This molecular quality control represents a form of mitochondrial retrograde signalling known as the UPR^mt^ (Haynes and Ron, 2010). The second level of mitochondria quality control is achieved through fusion and fission processes facilitated by the highly dynamic nature of mitochondria (Twig et al., 2008a,b). Damaged mitochondria can fuse with healthy organelles to restore the level of healthy components necessary for proper mitochondrial function. When the damage is excessive, the first two levels of quality control are overwhelmed mitochondria become depolarized and are targeted for recycling through a specific form of autophagy, termed mitophagy (Wang and Klionsky, 2011; Youle and Narendra, 2011). Components of this quality control pathway such as *PINK1* and *Parkin* have already been found mutated in PD or associated with increased susceptibility for PD like the mitochondrial protease *HTRA2* (reviewed in Rugarli and Langer, 2012).

The *PINK1* gene encodes a highly conserved serine–threonine kinase, mutations in which cause autosomal-recessive parkinsonism (Valente et al., 2004; Nuytemans et al., 2010). These mutations compromise the kinase activity or interfere with protein stability suggesting a loss-of-function mechanism in PD (Deas et al., 2009; Kawajiri et al., 2011; Thomas and Cookson, 2009). Further supporting this loss-of-function model, *Drosophila pink1* mutants show reduced life span, and degeneration of flight muscles and dopaminergic neurons (Clark et al., 2006; Park et al., 2006).

Deletion of *PINK1* in mice results in a subtle phenotype involving decreased dopamine levels motor deficits in aged mice (Gispert et al., 2009) and impaired synaptic plasticity (Kitada et al., 2007). A conditional KO of *PINK1* displays phenotypes reminiscent of early PD, including impaired gait, olfaction and serotonergic innervation (Glasl et al., 2012). Moreover, mitochondrial respiration is impaired in the *PINK1* KO mice (Gautier et al., 2008; Gispert et al., 2009).

A model that has now been established to study mitochondrial stress response and quality control is through overexpression of unfolded mitochondrial *ornithine transcarbamylase* (OTC). OTC is a mitochondrial enzyme involved in urea metabolism, normally only expressed in liver and small intestine and absent from transformed cells. Previous studies have established that accumulation of this protein in the mitochondrial matrix in an unfolded structure induced a *mitochondrial stress response* UPR^mt^, characterized by transcriptional up-regulation of mitochondrial chaperones and proteases (Zhao et al., 2002; de Castro et al., 2012).

Moreover we have recently reported the development of an *in vivo* model for mitochondrial dysfunction as a consequence of protein misfolding in *Drosophila*. In transgenic flies, the accumulation of abnormally folded proteins resulted in mitochondrial phenotypical alterations similar to those reported in *Drosophila* models of PD (Clark et al., 2006; Park et al., 2006).

Here we present a mouse model that reproduces cardinal features of the complex PD etiology. The model combines mitochondrial stress achieved through the expression of dOTC in the mitochondrial matrix together with impairment in the mitochondria quality control through genetic deletion of *PINK1*.

Using this model we show that the induction of mitochondrial stress in dopaminergic neurons results in neurodegeneration and motor impairment that is rescued by the administration of l-dopa. The process of neurodegeneration is accelerated by loss of *PINK1*.

## 2 Results

### 2.1 Generation of a system for the conditional expression of dOTC in mice

**Fig. 1:**
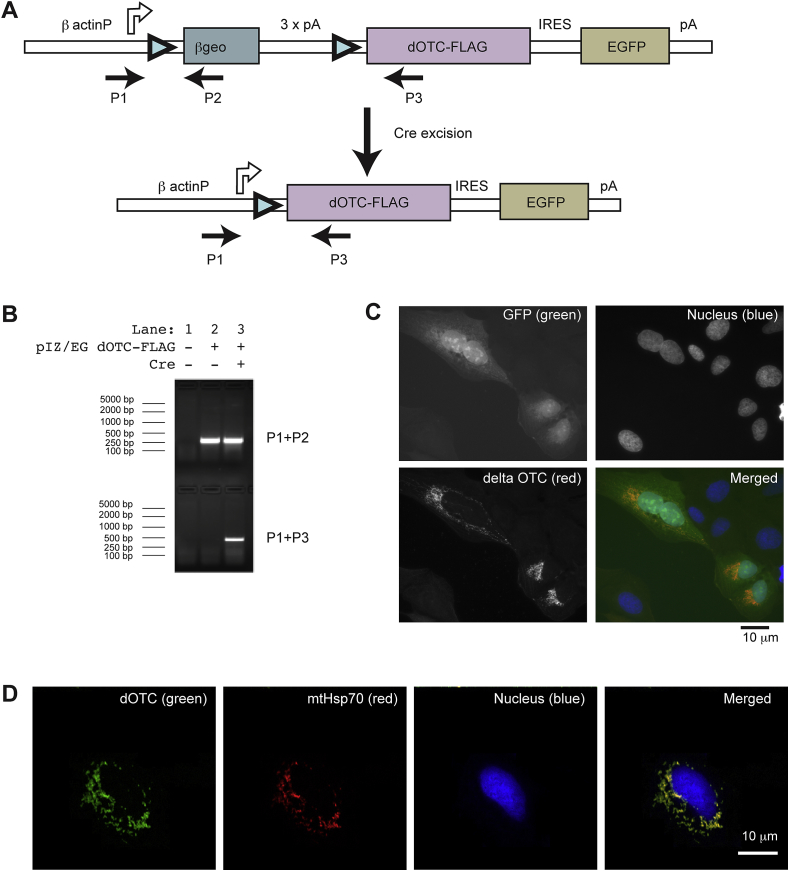
pZ/EG dOTC-FLAG expression construct. (A) The Z/EG construct for the expression of dOTC-FLAG consists of strong chicken β-actin promoter, directing the expression of a loxP-flanked (triangles) βgeo (lacZ/neomycin-resistance) fusion gene and three SV40 polyadenylation sequences. Following that, there is the coding sequence of dOTC-FLAG, that precedes an internal ribosomal entry site (IRES) and EGFP and a rabbit β globin polyadenylation sequence. In this configuration, βgeo is expressed before Cre excision whereas dOTC-FLAG and EGFP are expressed from a single mRNA after Cre excision. Indicated in the figure is the approximate position of oligonucleotide sequences used to detect the Cre-mediated recombination by PCR analysis (P1, P2, P3). (B) PCR-analysis of Cre mediated recombination. The recombination in U2OS cells was achieved by overexpressing the Z/EG dOTC-FLAG construct together with a plasmid expressing Cre recombinase under activation with tamoxifen. (C) Detection of EGFP in cells transfected with dOTC expression plasmid following Cre-mediated recombination. Expression of dOTC at protein level following recombination is shown by immunostaining (red). (D) dOTC (green) colocalization with mitochondria mtHsp70 (red) has been confirmed in U2OS cells transfected with dOTC in a vector for mammalian cells expression. (For interpretation of the references to colour in this figure legend, the reader is referred to the web version of this article.).

In previous work we used dOTC to develop a model for mitochondrial stress in the fruit fly, *Drosophila melanogaster* (de Castro et al., 2012). In order to extend these studies to a mammalian system, we made use of a system for the *in vivo* delivery of dOTC in mouse. To achieve this, we generated a dOTC-FLAG expression construct for *in vivo* studies based on the system described by Novak and colleagues (Novak et al., 2000) (Fig. 1A). This system enables the expression of dOTC by Cre mediated recombination. Additionally, cells where Cre-mediated recombination has taken place lose expression of a lacZ reporter transgene with concomitant activation of enhanced-GFP (EGFP) expression. We first demonstrated the efficient recombination of the transgene in U2OS cultured cells transfected with the dOTC expression construct either in the presence or absence of a Cre recombinase expression vector. The recombination at DNA level was confirmed by PCR (Fig. 1B), using two sets of primers as indicated in Fig. 1A and B. Next, we transfected cultured human cells with the transgene expression construct and confirmed at protein level that co-expression of Cre recombinase results in expression of dOTC with the EGFP reporter (Fig. 1C). Additionally we have validated the localization of the dOTC to the mitochondria by colocalization with mtHSP70 (Fig. 1D). We demonstrated elsewhere that dOTC-FLAG overexpressed *in vitro* in cell culture and *in vivo* in Drosophila accumulated in an unfolded state in the mitochondria (de Castro et al., 2012) and its capability to induce UPR^mt^ in these systems (Zhao et al., 2002; de Castro et al., 2012). Taken together this data validates our system for the controlled delivery of dOTC in transgenic mouse models with the purpose of inducing genetically a mitochondrial stress.

### 2.2 *In vivo* expression of dOTC in dopaminergic neurons

We first obtained transgenic mice containing the dOTC expression system. After Southern Blot analysis to confirm the successful integration of the gene, two transgenic lines containing a single integration (6-1C, 8-3A) were used for further characterization.

In order to induce mitochondrial stress in catecholaminergic neurons we crossed dOTC mice with a line that expresses the Cre recombinase under the control of the tyrosine hydroxylase promoter (Th-Cre line) (Savitt et al., 2005).

**Fig. 2:**
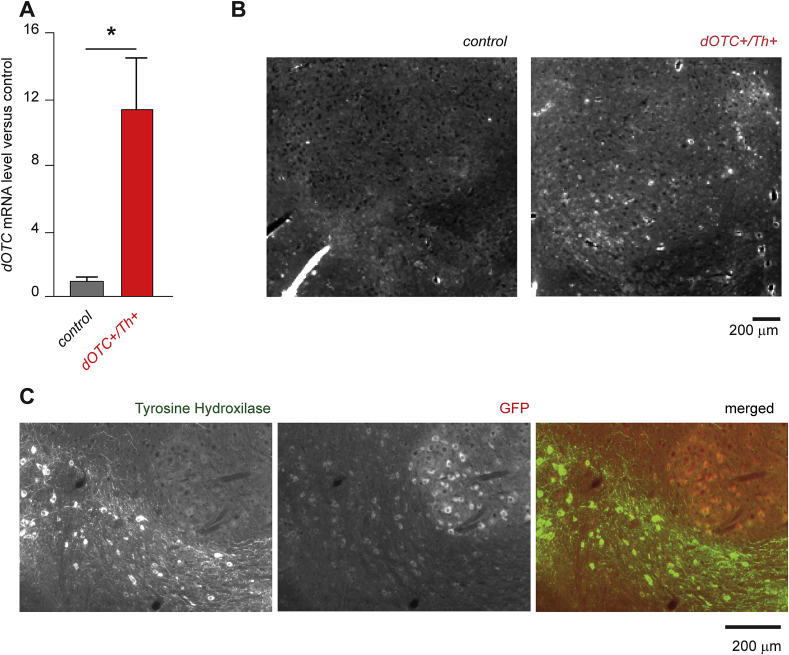
Expression of dOTC in Substantia Nigra. (A) The level of rat *OTC* mRNA in Substantia Nigra (SN) where the Cre recombination occurs under the tyrosine hydroxylase (Th) promoter. We detected a significantly higher mRNA level in the SN of the double transgenic mice. Statistical significance was analysed with Student *T*-test (*n* = 3) between the indicated groups. (B) EGFP fluorescence image was taken from brain slices (perfused and fixed in paraformaldehyde) with the same exposure for control and transgenic mice expressing dOTC-EGFP. The images covered an area of the brain including most of the Substantia Nigra and Ventral Tegmental Area. (C) Expression of dOTC in dopaminergic neurons confirmed using anti-GFP immunostaining (red) and by colocalization with tyrosine hydroxylase (green). (For interpretation of the references to colour in this figure legend, the reader is referred to the web version of this article.).

We then demonstrated, that dOTC is expressed in the tissue of interest. We measured the mRNA levels for the dOTC transgene in the Substantia Nigra (SN) where the Cre recombination occurs under the tyrosine hydroxylase promoter, detecting a significantly higher mRNA level in the double transgenic mice (Fig. 2A).

Additionally, we confirmed the correct expression of the transgene by measuring the fluorescence of the EGFP reporter that is co-expressed with dOTC (Fig. 2B). In order to increase the GFP signal and to verify the selective expression in dopaminergic neurons, we used anti-GFP staining (red) and colocalization with tyrosine hydroxylase stain (green) (Fig. 2C).

### 2.3 *PINK1* loss of function enhances the degenerative phenotype induced by mitochondrial stress

**Fig. 3:**
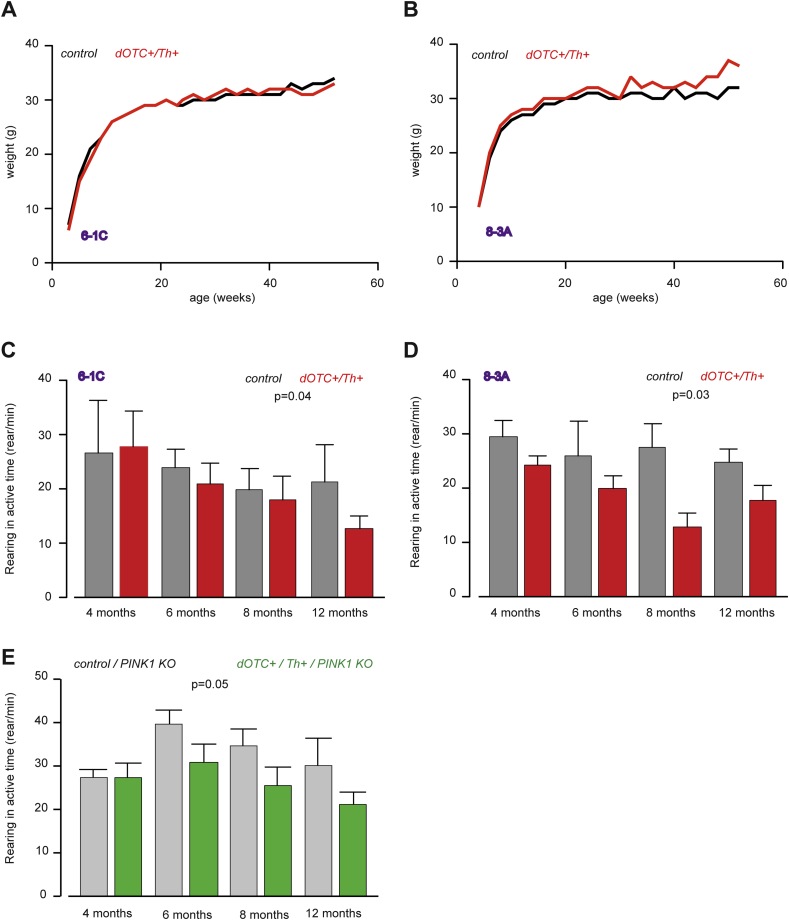
Motor behaviour characterization. (A, B) Growth curves of double transgenic mice from two independent lines with single integration of the dOTC transgene do not show a significant influence of the transgene expression on this parameter. (C, D) Motor ability assessed by rearing activity in ‘open field system’ is decreased in both lines. Two-way ANOVA for age and genotype show: panel C – statistically significant differences for genotype (line 6-1C, *p* = 0.04, *n* = 3–5) and no statistical difference for age; panel D – statistically significant differences for genotype (line 8-3A, *p* = 0.03, *n* = 3–5) and no statistical difference for age; (E) Motor behaviour analysis of triple transgenic overexpressing dOTC single integration from line 6-1C in *PINK1* KO background shows a more pronounced decrease of motor ability. Two-way ANOVA shows significant difference with genotype, *p* = 0.05, *n* = 4–9, and not significant difference with age.

To determine the consequences of enhanced stress caused by the expression of a mitochondrial protein in a misfolded state, the dOTC-expressing mice were characterized for both growth and motor behaviour. We did not observe any growth defects in dOTC expressing mice (Fig. 3A and B). However, in both lines there is a significant decrease in motor abilities as measured by reduced rearing movements (Fig. 3C and D).

One of the single integration lines (6-1C) has been used to study the effect of *PINK1* loss of function on the phenotype induced by the expression of dOTC (Fig. 3E). Due to the low probability to obtain all the desired genotypes in the same colony, the mice were bred on two different colonies: dOTC were crossed with Th-Cre on wild type *PINK1* background and on *PINK1* KO background. Therefore the comparisons were made between *dOTC+/Th+* versus control/WT, *dOTC+/Th+/PINK1 KO* versus control/*PINK1 KO*.

The motor ability analysis in the *PINK1* KO background shows a decrease in the rearing activity for the triple transgenic line as compared to *PINK1* KO. This appears to be accelerated as compared to the decrease in rearing activity induced by dOTC in the WT background. In the WT background the reduction in the rearing activity shows at one year of age while in the *PINK1* KO background it diminishes from six months.

**Fig. 4:**
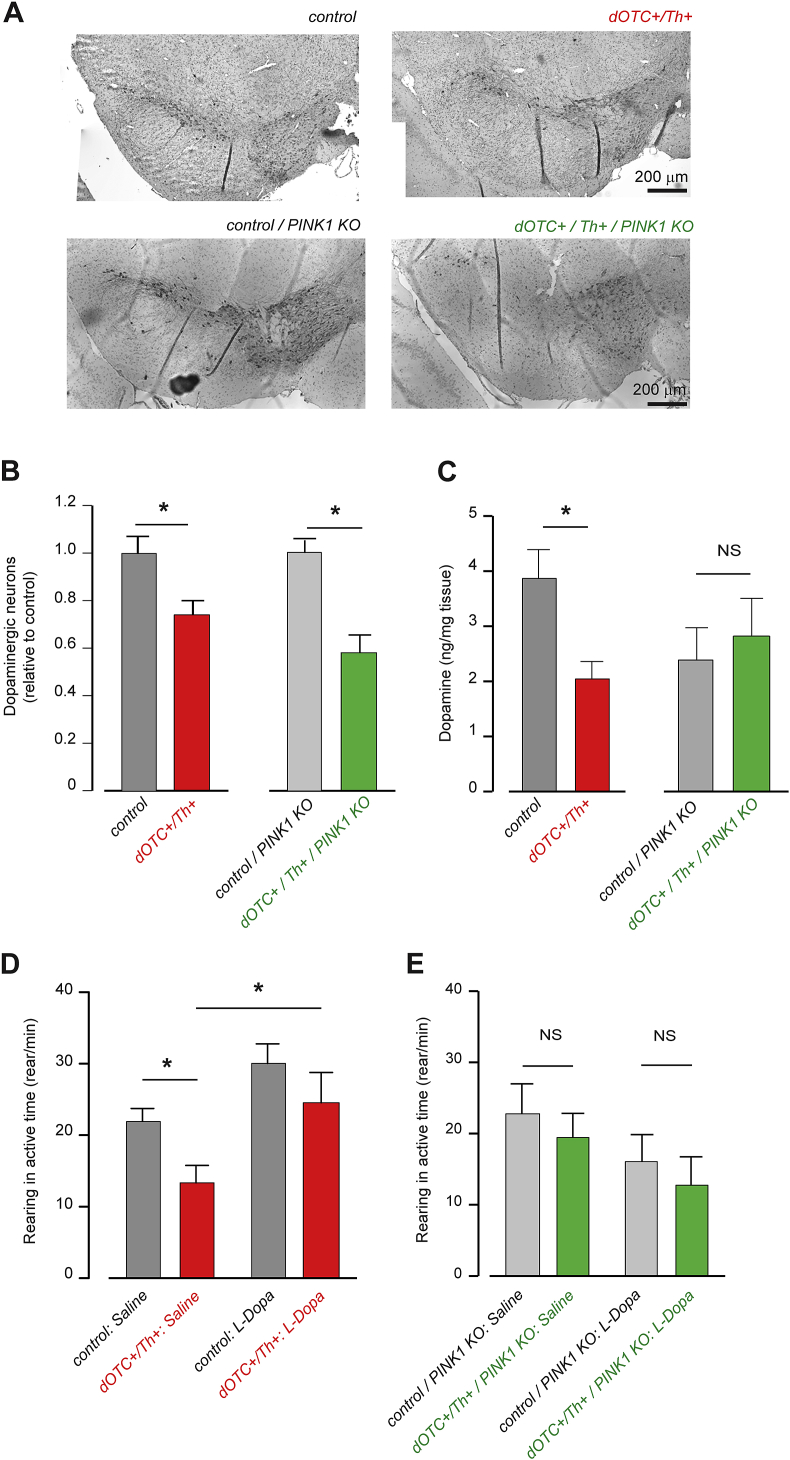
Neurodegeneration in the dopaminergic pathway. The neuronal cell death was assayed by immunohistochemistry of tyrosine hydroxylase positive neurons in brains of the indicated genotypes. Overexpression of dOTC induces cell death in the Substantia Nigra with a significant reduction in the dopaminergic neurons of 25% at one year of age (A, B) (*n* = 3). In the *PINK1* KO background this decrease is accentuated as shown by a more severe reduction in the number of Th positive neurons, to 40% (*n* = 3) (C) The level of dopamine in Substantia Nigra was analysed by HPLC measurements in tissue of the indicated genotype. The dopamine content is reduced in the double transgenic in WT background, versus control. Overexpression of dOTC in *PINK1* KO background does not decrease the dopamine level as compared to *PINK1* KO control. (D) Pharmacological rescue of behavioural deficit with l-dopa shows increased activity in a population of mice from double transgenics in both control and dOTC overexpressing mice. The data presented here are from responsive mice (*n* = 4 WT, *n* = 3 *PINK1* KO). (E) For triple transgenic mice with dOTC expressed in *PINK1* KO background there is no response reflected in rearing activity changes following l-dopa treatment. (*n* = 8 for each WT and PINK1 KO). The analysis was performed with Student *T*-test between the indicated groups.

We then characterized the transgenic mice from one of the lines (6-1C) for cell death and dopamine content in the Substantia Nigra. The tyrosine hydroxylase (Th) positive cell counts show 25% decrease in neuronal population in *dOTC+/Th+* at one year of age (Fig. 4A and B). Similarly with the motor dysfunction trait, loss of *PINK1* function accelerates this phenotype. Thus, in *PINK1* KO background overexpression of dOTC induces a cell loss of about 40% in SN (Fig. 4A and B). We have then measured the level of dopamine in the Substantia Nigra by HPLC. *PINK1* KO mice are known to present a reduction in dopamine levels that becomes significant from 18 months of age in spite of no dopaminergic cell loss being detected (Gispert et al., 2009). In our experimental set-up the *PINK1* KO strain versus WT presented a reduction in the dopamine levels reported to the mass of tissue at 12 months of age, but this was not significant to 95% with a *p* > 0.05 in the Student *T*-test. Due to the breeding protocol in two separated colonies (WT respectively *PINK1* KO background) we have presented the data comparing the *dOTC+*/*Th+* transgenics to littermates control within the individual colonies. The dOTC overexpressed in WT background demonstrates a significant decrease in the dopamine levels versus control. Interestingly in the *PINK1* KO background overexpression of dOTC does not induce a further reduction in the dopamine levels (Fig. 4C).

In order to assess whether the parkinsonian phenotype we have achieved in this transgenic mice is dependent on l-dopa, we performed a pharmacological rescue experiment analysing the motor behaviour after l-dopa injection. Injection of l-dopa induced an increase in the rearing movement in 50% of controls and in 60% of double transgenics (Fig. 4D). The increase in motor ability is significant suggesting that motor dysfunction caused by mitochondrial stress is rescued by l-dopa. Most strikingly the injection of l-dopa in the *PINK1* KO background does not improve the motor behaviour in any of the mice assessed (Fig. 4E). This suggests that the dopaminergic signalling is impaired in the transgenic mice presenting both dOTC induced mitochondrial stress and *PINK1* loss of function.

## 3 Discussion

Sporadic PD appears to have a complex multifactorial etiology with variable contributions from environmental factors and genetic susceptibility.

It is now recognized that Parkinson's disease is a multisystem neurodegenerative disorder that affects multiple areas of the brain (Braak et al., 2003) with motor symptoms appearing when greater than 50% of the dopamine neurons in the Substantia Nigra are lost. Modelling prodromal symptomatology of PD appears possible using transgenic mice with loss of function in *PINK1*, *PARKIN*, *DJ-1* genes or gain of function in *LRKK2*, and *α-synuclein* [reviewed in Smith et al. (2012)], genes found mutated in PD. The early symptomatology from these models offers the possibility of targeting dysfunctional pathways, prior to severe neuronal loss when drug treatments appear to improve symptoms but not to target the disease progression.

Mutations in *PINK1* serine–threonine kinase are the second most common cause of autosomal recessive parkinsonism after mutations in the *PARKIN* gene (Valente et al., 2004). About 30 pathogenic mutations have been associated with the disease [reviewed in Nuytemans et al. (2010)]. Deletion of *PINK1* in mice does not result in an overt phenotype. The mice display only subtle deficits, which differ slightly between different loss-of-function models, but converge to give a whole picture of a prodromal model of PD. Thus the phenotype present only minor decrease in total dopamine levels in very old mice (Gispert et al., 2009), impaired synaptic plasticity in the striatum but no loss of dopaminergic neurons in Substantia Nigra (Kitada et al., 2007) respectively a minor loss of dopamine cells in a more recent conditional model of *PINK1* loss-of-function (Glasl et al., 2012). Mitochondrial respiration is impaired in the striatum of *PINK1* KO and respiration deficits can be induced in the cortex by cellular stress (Gautier et al., 2008). The mobilization of reserve pool synaptic vesicles at the neuromuscular junction of *pink1* deficient flies is impaired during rapid stimulation due to synaptic ATP depletion, indicating that synaptic activity cannot be maintained under increased energy demand in *pink1* deficient neurons (Morais, 2009).

There is strong published data from *in vitro* and *Drosophila* models linking *PINK1* to mitochondrial function and mitochondrial quality control together with Parkin (reviewed in Pilsl and Winklhofer, 2012). However, the role of the *PINK1*/*Parkin* pathway *in vivo* needs to be studied further particularly due to the fact that recent work from Larsson and colleagues could not demonstrate an *in vivo* role for Parkin in mitochondrial quality control in a mouse model of Parkinsonism based on dopaminergic neuron-specific loss of the mitochondrial transcription factor A (TFAM) (Van Laar et al., 2011; Sterky et al., 2011).

The data presented here demonstrates for the first time in an *in vivo* model that *PINK1* loss of function is capable to accelerate neurodegenerative phenotypes induced by mitochondrial stress. We made use of a model of mitochondrial stress induction through the expression of a protein in the mitochondrial matrix that has been previously characterized both *in vitro* and *in vivo* (Zhao et al., 2002; de Castro et al., 2012). Our data indicates that the accumulation of a misfolded protein in the dopaminergic neurons is capable of inducing neurodegeneration and motor behaviour impairment that illustrates a parkinsonian phenotype. This associates with l-dopa responsiveness validating the model as a model of Parkinsonism. We hypothesise further that increased expression of dOTC would be able to accelerate the parkinsonian phenotype that we have obtained here and would produce a model that can be used in pharmacological studies for Parkinson's treatments.

Our results show that *PINK1* loss of function accelerates the motor deficit and the dopaminergic degeneration induced by overexpression of dOTC in the mitochondria. First, the motor behaviour impairment although mild, as expected from a prodromal model of PD, appears faster in the *PINK1* KO background than in the WT. *PINK1* KO mice did not present a loss of dopaminergic neurons up to one year of age, similarly with the phenotypes reported in the literature and reviewed here. However, the *PINK1* loss of function appears to accentuate the loss of dopaminergic neurons determined by the dOTC induced mitochondrial stress. Interestingly, overexpression of dOTC in the *PINK1* KO background does not decrease the dopamine levels as compared to the *PINK1* KO control. These results suggest that loss of dopamine production might occur in the same neuronal population or through the same mechanistic pathway for both *PINK1* loss of function and dOTC expression.

Most strikingly, the loss of *PINK1* compromised the rescue of the motor deficit induced by dOTC mitochondrial stress with l-dopa. Previous published data mention that *PINK1* loss of function results in impaired dopaminergic signalling manifested in low dopamine release from nigrostriatal terminals and consequent reduction in activation of specific postsynaptic receptors. This is accompanied by defects in corticostriatal electrophysiological properties (Kitada et al., 2007). The fact that l-dopa injection does not appear to improve the motor deficit caused by mitochondrial stress in *PINK1* KO background supports the hypothesis that the model presented here demonstrates a deficit in transmission of dopaminergic signalling.

In spite of the mild phenotypes that *PINK1* KO mice show, this model has established itself as a useful prodromal model of PD (Smith et al., 2012). Here we make use of it to demonstrate that *PINK1* loss of function contributes to parkinsonian neurodegeneration. Moreover the combination of mitochondrial stress and *PINK1* loss of function demonstrates for the first time that PD has a multifactorial etiology where genetic and environmental factors might synergize to increase neurodegeneration.

## 4 Materials and methods

### 4.1 Expression of dOTC

The deletion mutant dOTC (30-114) fused to a carboxy-terminal FLAG tag sequence was cloned into the conditional Z/EG expression vector (Novak et al., 2000).

Recombination was assayed using a PCR strategy with primers P1 (5′-TCTGCTAACCATGTTCATGCC-3′), P2 (5′-ATGTGCTGCAAGGCGATTAAG-3′) and P3 (5′-TCTGACAGTCCGTTGACAATTG-3′). The recombination was tested in U2OS cells by transfection (with Effectene) of the Z/EG dOTC-FLAG expression vector together with the Cre expression vector pCAGGS_Cre-ER (a gift from Prof. Catrin Pritchard, University of Leicester). Expression of Cre was induced 24 h after transfection by addition of 4-hydroxy-tamoxifen (1 mM) for 48 h. The cells were than fixed and extracted with 3% PFA in microtubule stabilizing buffer (Moisoi et al., 2002) and processed for immunofluorescence with the antibody against OTC (Sigma). For colocalization of dOTC with the mitochondria, dOTC was overexpressed in U2OS using a mammalian expression vector, pcDNA3-dOTC, and the cells were fixed and processed for immunofluorescence with the indicated antibodies (OTC respectively mtHSP70).

### 4.2 Animal husbandry

Animal husbandry and experimental procedures were performed in full compliance with the United Kingdom Animal (Scientific Procedures) Act 1986.

The dOTC lines have been produced and characterized for the integration of the gene by GENEOWAY, Lyon, France. The mice used in these experiments have been backcrossed 5–6 times to a C57/B6 background.

The *PINK1* KO mice have been obtained from LEXICON GENETICS and have been described previously (Wood-Kaczmar et al., 2008). They were fully backcrossed on C57/B6 background (more than ten times).

The Th-Cre line was from Jackson's laboratory and they are bred on a C57/B6 background.

In order to obtain the desired genotypes the mice were bred on two different colonies: dOTC were crossed with Th-Cre on wild type *PINK1* background and on *PINK1* KO background. For the experiments presented here we used males.

### 4.3 Mice genotyping

The genotyping of dOTC mice was performed within the EGFP locus with the primers: oIMR0042(CTAGGCCACAGAATTGAAAGATCT), oIMR0043(GTAGGTGGAAATTCTAGCATCATCC), oIMR0872(AAGTTCATCTGCACCACCG), oIM1416(AGATGGTGCG). The top band at 324 bp is a positive control band and the bottom band at 173 bp represents the EGFP locus genotype.

The ThCre mice were genotyped using the primers:

Th-Cre(+) (AAATGTTGCTGGATAGTTTTTACTGC)
Th-Cre(−) (GGAAGGTGTCCAATTTACTGACCGTA)

This genotyping protocol provides a single band for the mutant locus at 300 bp.

The *PINK1 KO* genotype was performed using the primers:

EB0088-26 (CTGCCCTCAGGGTCTCTAATGC),
EB0088-27 (GGAAGGAGGCCATGGAAATTGT),
Neo3a (GCAGCGCATCGCCTTCTATC)

The top band at 296 bp genotypes the wt locus, the bottom band at 193 bp genotypes the mutant locus.

### 4.4 Behavioural testing

Locomotor activity was assessed using a computer-controlled photocell-based system (Linton Instruments, UK) as described previously (Moisoi et al., 2009). The activity has been recorded every 10 min over 1 h and the result is given as average of the 6 measurements. The parameter reported is ‘Rearing activity’ calculated as number of rearings in active time.

### 4.5 Pharmacological rescue

Transgenic mice and control littermates were injected intraperitoneal with saline control (0.9% NaCl) or methyl levodopa hydrochloride 25 mg/kg with benserazide 6.5 mg/kg (Sigma) in saline solution. Behavioural testing was performed 40 min after the injection.

### 4.6 Histology, immunohistochemistry and immunofluorescence of brain tissues

In order to confirm the protein overexpression the brains of 2 months old mice were perfused and fixed in 4% paraformaldehyde. Coronal sections were cut at 10 μm thickness. For GFP signal, images were taken at the same exposure for both control and transgenic mice. In order to check the localization of the GFP signal in the dopaminergic neurons, the sections were processed for immunofluorescence using an anti-GFP (Roche) and anti-tyrosine hydroxylase antibodies.

For tyrosine hydroxylase staining, and dopaminergic cell counts, brains were harvested from one-year mice old and processed as described previously (Moisoi et al., 2009). Dopaminergic neurons were visualized by immunostaining with anti-tyrosine hydroxylase antibody using the DAKO duet system (DAKO K0492) with 3,3, diaminobenzidine (DAB) as the chromagen according to the manufacturer's instruction. The immunostained sections were counterstained with hematoxilin.

### 4.7 Neuronal quantitation

Counts of dopaminergic neurons (tyrosine hydroxylase positive) were performed in the area of Substantia Nigra and Ventral Tegmental Area. Whole brain coronal sections were reconstituted from digital images acquired with a 10× objective using the Photomerge tool in Adobe Photoshop CS3. Counts of tyrosine hydroxylase cells were performed using stereologic methods as described (Shin et al., 2011).

### 4.8 HPLC measurement of dopamine

For sample preparation, Substantia Nigra of one-year old males with the indicated genotypes was dissected and quickly frozen in liquid nitrogen. The tissue was weighed and homogenized in 200 μl chilled 0.01 M perchloric acid using a motorized, hand-held tissue homogenizer. The chilled homogenates were filtered through a low-binding Durapore (0.22 μm) PVDF membrane using Ultrafree-MC centrifugal devices. The dopamine measurement was performed immediately after the sample preparation. Supernatant fluid was eluted at a flow rate of 50 μl/min through a 150 × 1.0 mm C18 column (ALF-115, ANTEC). As recommended by the manufacturer instructions the mobile phase contained: 50 mM phosphoric acid, 50 mM citric acid, 8 mM NaCl, 0.1 mM EDTA, 10% methanol, 350 mg/l OSA (pH 3.2). Analysis was performed using an Alexys LC-EC system equipped with a DECADE II electrochemical detector (ANTEC). The level of dopamine was reported to the mass of tissue and the values are presented as ratio to control.

### 4.9 Quantitative real-time RT-PCR

Quantitative RT-PCR was performed on an Mx4000 (Stratagene) real-time cycler using the QuantiTect SYBR Green RT-PCR system (QIAGEN). Primers for rat OTC were obtained from QIAGEN (QuantiTect Primer Assays). The relative transcript levels of the target genes were normalized against mouse GAPDH mRNA levels. Quantification was performed using the comparative Ct method (Schmittgen and Livak, 2008).

### 4.10 Statistical analysis

Data are presented as mean values, and error bars indicate ±SD. Inferential statistical analysis was performed using the Prism and StatMate software packages (www.graphpad.com). The significance level is indicated as * for *p* ≤ 0.05 and NS for *p* > 0.05.
